# Porosity of temporary denture soft liners containing antifungal agents

**DOI:** 10.1590/1678-775720160092

**Published:** 2016

**Authors:** Jozely Francisca Mello Lima, Janaína Gomes Maciel, Juliana Hotta, Ana Carolina Pero Vizoto, Heitor Marques Honório, Vanessa Migliorini Urban, Karin Hermana Neppelenbroek

**Affiliations:** 1 Universidade de São Paulo, Faculdade de Odontologia de Bauru, Departamento de Prótese, Bauru, SP, Brasil; 2 Universidade Estadual Paulista, Faculdade de Odontologia de Araraquara, Departamento de Materiais Odontológicos e Prótese, Araraquara, SP, Brasil; 3 Universidade de São Paulo, Faculdade de Odontologia de Bauru, Departamento de Odontopediatria, Ortodontia e Saúde Coletiva, Bauru, SP, Brasil; 4 Universidade Estadual de Ponta Grossa, Departamento de Odontologia, Ponta Grossa, PR, Brasil

**Keywords:** Denture stomatitis, Denture liners, Porosity, Anti-infective agents

## Abstract

Incorporation of antifungals in temporary denture soft liners has been recommended for denture stomatitis treatment; however, it may affect their properties.

**Objective:**

To evaluate the porosity of a tissue conditioner (Softone) and a temporary resilient liner (Trusoft) modified by minimum inhibitory concentrations (MICs) of antifungal agents for *Candida albicans* biofilm.

**Material and Methods:**

The porosity was measured by water absorption, based on exclusion of the plasticizer effect. Initially, it was determined by sorption isotherms that the adequate storage solution for specimens (65×10×3.3 mm) of both materials was 50% anhydrous calcium chloride (S50). Then, the porosity factor (PF) was calculated for the study groups (n=10) formed by specimens without (control) or with drug incorporation at MICs (nystatin: Ny-0.032 g, chlorhexidine diacetate: Chx-0.064 g, or ketoconazole: Ke-0.128 g each *per* gram of soft liner powder) after storage in distilled water or S50 for 24 h, seven and 14 d. Data were statistically analyzed by 4-way repeated measures ANOVA and Tukey's test (α=.05).

**Results:**

Ke resulted in no significant changes in PF for both liners in water over 14 days (p>0.05). Compared with the controls, Softone and Trusoft PFs were increased at 14-day water immersion only after addition of Ny and Chx, and Chx, respectively (p<0.05). Both materials showed no significant changes in PF in up to 14 days of S50 immersion, compared with the controls (p>0.05). In all experimental conditions, Softone and Trusoft PFs were significantly lower when immersed in S50 compared with distilled water (p<0.05).

**Conclusions:**

The addition of antifungals at MICs resulted in no harmful effects for the porosity of both temporary soft liners in different periods of water immersion, except for Chx and Ny in Softone and Chx in Trusoft at 14 days. No deleterious effect was observed for the porosity of both soft liners modified by the drugs at MICs over 14 days of S50 immersion.

## INTRODUCTION

Denture stomatitis (DS), an oral lesion found in approximately 65% of removable denture wearers, has been associated with multiple etiologic factors^13^. The systemic factors include physical impairment, allergies, age, gender, tobacco, endocrinopathies, nutritional deficiencies, wide spectrum antibiotics, immunosuppressants, antineoplastic therapies, and immune system disorders^13^. Also, local factors associated with the dentures have been considered determinant in the development or maintenance of DS, especially the following: presence of biofilm^22^, local trauma caused by the dentures, mainly old and ill-fitting dentures^13^, hyposalivation^13^, continuous utilization of dentures^9,13,22^, and decreased salivary pH^13^. Despite the multifactorial etiology, the involvement of *Candida* species, with emphasis to *Candida albicans,* is considered the main factor related to the establishment and persistence of DS^13^.

Considering the relevant role played by local factors and the *Candida* infection in DS, the incorporation of antimicrobial agents in temporary soft denture liners has been suggested^26,27^. The resilient materials modified by drugs may act as a potential therapy for DS treatment for several reasons: 1) the contact between the prosthetic biofilm and infected tissues is eliminated, preventing reinfection through the denture base; 2) different from conventional topical antifungals, the gradual release of antimicrobials through the liners is able to achieve an effective therapeutic concentration in infected sites, even under the diluent effects of saliva/swallowing and tongue movements; 3) therapy is based on utilization of the relined denture, eliminating the necessity of patient compliance; 4) relining with the soft material allows readaptation of the denture base to supporting tissues, minimizing the tissue trauma that worsens the infection and provides comfort to the patient; 5) the period of utilization of a denture relined with a short-term resilient liner is short, similar to the period of treatment with conventional topical antifungal (14 days), with the advantage of recovering the injured tissues and preventing accumulation of biofilm until denture replacement or relining with long-term materials^26,27^.

Conversely, the modification of temporary soft liners by drugs at commercially available concentrations may affect their structural, physical, and mechanical properties^1,26,28–30^, which may impair the clinical performance of these materials during their lifespan. Aiming to maintain the properties of modified materials, a previous study^6^ aimed to determine drug concentrations lower than those commercially available, yet simultaneously effective for fungal inhibition. Over 14 days, the minimum inhibitory concentrations (MICs) able to inactivate the *C. albicans* biofilm (SC5314) in temporary soft liners analyzed (Trusoft and Softone) were achieved with nystatin (polyene antifungal antibiotic), chlorhexidine diacetate (broad-spectrum antimicrobial agent), and ketoconazole (azole antifungal drug)^6^. However, the effects of the addition of these drugs MICs on the properties of modified resilient liners are still unknown.

Among the several properties that must be analyzed in a polymer, porosity presents great relevance, also from a clinical standpoint, since this is a complex phenomenon of multifactorial origin that may even weaken denture bases^23^. Microporosities present on denture surfaces may also facilitate the adhesion and proliferation of microorganisms, interfering with the mechanical and chemical procedures for denture cleaning^24^. Resilient materials are considered more susceptible to microbial adhesion than thermo-polymerized acrylic resins, since they present higher capacity to interact with microorganisms because of their surface texture and physicochemical affinity with such materials^24^.

The presence of pores on denture base materials is an undesirable characteristic and thus it has been evaluated by several methods, including analysis of the association of porosity with the volume of water absorbed by the polymer^8^. Despite the importance of the plasticizer effect of water on the clinical performance of acrylic denture base materials, this factor has not been considered in related studies, which also employed only water storage to evaluate the porosity of specimens^8^. An adequate analysis of porosity requires a method of water sorption that takes into account the elimination of the plasticizer effect of water in the polymer structure. The storage of acrylic resin in salt-saturated aqueous solutions, which allows variation of water activity, provides an estimate of the solution in which the plasticizer effect of water would not occur (optimal water activity)^25^. Considering these aspects, a method for evaluation of porosity by water sorption based on determination of an adequate storage solution (*S*) of denture base resins has been recently validated^23^. Through this method it was observed that, in distilled water, the plasticizer effect was not eliminated, and the optimal storage solution varied according to the type of denture base resin employed, yet it did not vary according to the specimen shape for the same material^23^.

The effect of addition of drugs on the porosity of temporary soft liners is not known, which is relevant, since this modification has proven to be effective for inhibition of *C. albicans* biofilm, the main pathogen involved in DS. Therefore, this study evaluated the effect of incorporation of antifungals at MICs on the porosity of short-time resilient liners during their lifespan, employing the method using water sorption based on determination of the optimal storage solution, in which the plasticizing effect was excluded. The null hypothesis investigated was that the addition of drugs would not adversely affect the porosity of temporary liner after different storage periods in distilled water or optimal storage solution determined for each material.

## MATERIAL AND METHODS

### Determination of optimal storage solution (S)

Rectangular specimens (65×10×3.3 mm) were achieved according to specification #1567 of the International Organization for Standardization^17^, using a stainless steel metallic mold. The ISO standard for denture base resins was selected because there is no specification on the dimensions of specimens for analysis of porosity of resilient liners. Also, no method has been defined by ISO for evaluation of porosity in this type of material.

Initially, to determine the *S* of each material, a total of 80 non-modified specimens (without drug) were achieved, being 40 for temporary resilient liner Trusoft (Bosworth Company, Skokie, IL, USA) and 40 for the tissue conditioner Softone (Bosworth Company, Skokie, IL, USA). To achieve each specimen, the material was proportioned and mixed following the manufacturer's instructions and poured in a stainless steel mold (65×10×3.3 mm), positioned between two glass slides until plasticization (6-7 min).

After processing, the specimens were dried in a vacuum desiccator at 37±0.5°C and were subjected to desorption. They were weighed daily in a digital analytical balance (UMark 210 A, BEL Engineering, Monza, MI, Italy) until a constant mass was obtained, indicating a state of equilibrium. Specimen mass was considered stable when the difference between the mean of each evaluation period was lower than 0.0002 g over 24 h^23^. Recording of these weights allowed achievement of the respective temporal curves of desorption. After the state of equilibrium was established, 10 specimens of each material were individually immersed in one of the different solutions corresponding to the different values of water activity: anhydrous calcium chloride solutions at 25%, 50%, 75%, and distilled water. The specimens were kept at 37±0.5°C, removed from the solutions daily, carefully dried with absorbent paper and weighed until the state of equilibrium was reached, as previously described.

To determine an *S* for each material, based on exclusion of the plasticizing effect, sorption isotherms were obtained. On these isotherms (y-axis: water absorption; x-axis: storage solution), the region where a positive deviation of the curve was observed indicates the presence of the plasticizing effect of water on the material^25^.

For both resilient materials and regardless of the storage solution, the periods of water desorption and absorption were eight and nine days, respectively. The mean values of equilibrium water uptake were used to obtain the sorption isotherms to determine *S*. The optimal storage solution for porosity evaluation of both soft liners was anhydrous calcium chloride solution at 50% (*S*50).

### Calculation of the porosity factor (PF)

After determination of *S*50 for the materials Trusoft and Softone, specimens were achieved for measurement of the porosity factor (PF). These specimens were fabricated following the same protocol described above for specimens employed for determination of *S*, yet modified or not by the following drugs: nystatin-Ny (Pharma Nostra Comercial Ltda., Rio de Janeiro, RJ, Brazil), chlorhexidine diacetate-Chx (Acros Organics, New Jersey, NJ, USA), and ketoconazole-Ke (Galena Química e Farmacêutica Ltda., Campinas, SP, Brazil). The drugs were used in powder form to allow easy incorporation into the soft liner powder^6,29^.

During specimen fabrication, the drugs were added to the soft liner powders at MICs for *C. albicans* biofilm (0.032 g for Ny, 0.064 g for Chx, and 0.128 g for Ke *per* g of resilient material powder)^6^. The liquid was added to the powder (material and drug) and mixed according to the manufacturer's instructions. A control group without addition of drugs was obtained for both materials. The same procedures described for weighing until achievement of dry mass stabilization were performed. Then, after equilibrium, the specimens were immersed for 24 h, seven and 14 days^6,28^ in *S*50 or pure distilled water for calculation of PF. Ten repetitions were performed for each experimental condition, adding up to 160 specimens. The PF was calculated based on studies that evaluated porosity by water sorption^8,23^. Two weighing stages were thus performed: initial weighing was obtained for the dried specimen, and final weighing for the wet specimen, after storage in *S*50 and also for the condition of storage in distilled water. Since this analysis of porosity is based on the Archimedes' Principle, in each experimental condition (dried specimen and wet specimen), an additional weighing was performed for the specimen immediately immersed in water. The PF was calculated based on the following arithmetic equations^8,23^.


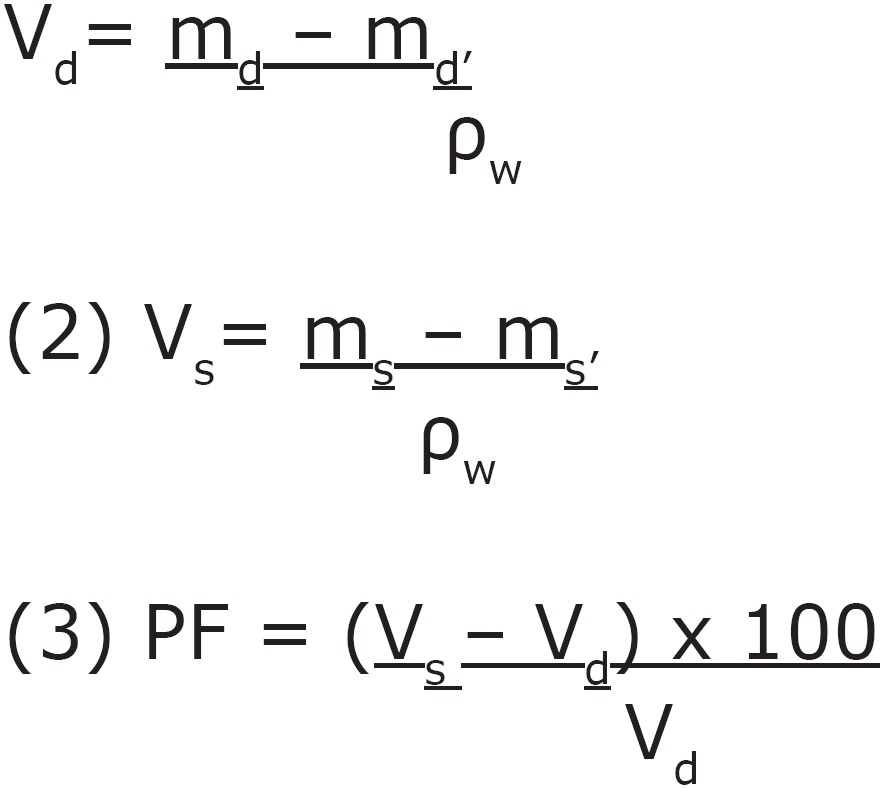


Where: V_d_= dried specimen volume; m_d_= mass of dried specimen in air; m_d_'= mass of dried specimen in water; ρ_w_= density of water; V_s_= volume of the specimen saturated with water; m_s_= mass of saturated specimen in air and m_s_'= mass of saturated specimen in water. In the first and second equations, the volumes were determined, using the following known values obtained by weights and the ρ_w_= 1000 Kg/m^3^.

Since the same specimens were analyzed at each storage period (24 h, seven and 14 days), PF data were statistically analyzed by 4-way repeated measures ANOVA (“material,” “drug,” “solution”, and “time”) followed by Tukey's test (α=0.05).

## RESULTS

Table 1 presents the mean values and standard deviations of PF (%) and the statistical significance for all experimental conditions analyzed.

**Table 1:** Porosity factor means (%) ± standard deviations for the experimental conditions

Material	Solution	Drug	Time	Mean±SD
		Ct	24 h	1.3056±0.6263 ^j,k,l,m,n,o,p,q^
	water	Ct	7 d	2.3636±0.7355 ^o,p,q^
		Ct	14 d	5.4969±1.1767 ^r^
		Ny	24 h	1.0245±0.7547 ^h,i,j,k,l,m,n,o,p^
	water	Ny	7 d	2.8333±0.9002 ^p,q,r^
Softone		Ny	14 d	8.5627±0.8821 ^s^
		Chx	24 h	1.3321±0.6453 ^k,n,o,p,q^
	water	Chx	7 d	1.5467±3.4518 ^l,m,n,o,p,q^
		Chx	14 d	11.5219±2.0875 ^t^
		Ke	24 h	1.6519±1.0175 ^m,n,o,p,q^
	water	Ke	7 d	2.7116±0.7885 ^p,q^
		Ke	14 d	5.1151±1.3841 ^r^
		Ct	24 h	0.2857±0.1101 ^e,f,g,h,i,j,k,l,m,n,o,p^
	water	Ct	7 d	0.4307±0.2023 ^f,g,h,i,j,k,l,m,n,o,p^
		Ct	14 d	1.4310±0.8509 ^j,k,l,m,n,o,p,q^
		Ny	24 h	-0.9898±0.8831 ^d,e,f,g,h,^i
	water	Ny	7 d	-1.8326±0.8159 ^b,c,d,e,f,g^
Trusoft		Ny	14 d	-0.0185±0.3211 ^e,f,g,h,i,j,k,l,m,n,o,p^
		Chx	24 h	1.7215±1.1011 ^m,n,o,p,q^
	water	Chx	7 d	-0.3818±1.0176 ^d,e,f,g,h,i,j,k,l,m,o^
		Chx	14 d	6.1581±0.6295 ^r,s^
		Ke	24 h	0.9929±0.8091 ^h,l,m,n,o,p,q^
	water	Ke	7 d	-1.2857±0.7547 ^c,d,e,f,g,i,j,k^
		Ke	14 d	1.6355±0.4346 ^l,m,n,o,p,q^
		Ct	24 h	0.5918±0.4720 ^g,j,k,l,m,n,o,p^
	S50	Ct	7 d	-1.4705±1.0535 ^c,d,e,f, h,i^
		Ct	14 d	0.0872±0.0998 ^e,f,g,h,i,j,k,l,m,n,o,p^
		Ny	24 h	-0.0746±0.0531 ^e,f,g,h,i,j,k,l,m,n,o^
	S50	Ny	7 d	-2.9376± 1.6410 ^b,c,d^
Softone		Ny	14 d	-1.1049±0.9633 ^c,d,e,f,g,h,i,j,k,l^
		Chx	24 h	0.3866±0.3692 ^f,g,h,i,j,k,l,m,n,o,p^
	S50	Chx	7 d	-3.0844±1.6763 ^b,c,d^
		Chx	14 d	-1.1004±0.9661 ^c,d,e,f,g,h,i,j,k,l^
		Ke	24 h	0.1614±0.1554 ^f,g,h,i,j,k,l,m,n,o,p^
	S50	Ke	7 d	-2.4322±1.0137 ^b,c,d,e^
		Ke	14 d	-0.8200±0.6757 ^d,e,f,g,h,i,j,k,l,m^
		Ct	24 h	-0.0789±0.0143 ^e,f,g,h,i,j,k,l,m,n,o^
	S50	Ct	7 d	-0.5098±0.1510 ^d,e,f,g,h,i,j,k,l,m,n^
		Ct	14 d	0.5663±0.1914 ^f,g,h,i,j,k,l,m,n,o,p^
		Ny	24 h	-1.5900±0.3926 ^c,d,e,f^
	S50	Ny	7 d	-1.9904±0.4427 ^a,b,c,d,e^
Trusoft		Ny	14 d	-0.7907±0.3912 ^d,e,f,g,h^
		Chx	24 h	0.6542±0.2699 ^f,g,h,i,j,k,l,m,n,o,p^
	S50	Chx	7 d	-0.3440±0.1395 ^d,e,f,g,h,i,j,k,l,m,o^
		Chx	14 d	1.9363±1.2703 ^n,p,q^
		Ke	24 h	-0.11 00±0.1033 ^e,f,g,h,i,j,k,l,m,n,o^
	S50	Ke	7 d	-1.7586±1.0185 ^c,d,e,f,g^
		Ke	14 d	1.0845±0.8091 ^h,i,j,k,l,m,n,o,p,q^

Vertically, different lower case letters indicate significant differences between the experimental conditions (solution, drug, and time) (P<0.05)

The results of ANOVA demonstrated statistically significant differences between resilient materials, between drugs incorporated in the materials, between evaluation periods, between storage solutions of specimens, and between all possible interactions of all factors (2 by 2, 3 by 3, and all to each other) (p<0.05).

Comparison between groups modified by drugs in each evaluation period showed that, compared with the control, the addition of Ke did not cause significant change in the PF for Softone immersed in water up to 14 days (p>0.05). Concerning the control, modification by Ny and Chx caused significant increase in the PF of Softone at 14 days of water immersion (p<0.05), and this difference was not observed in periods of 24 h and seven days (p>0.05) (Table 1). Regardless of the addition of drugs, water immersion for 14 days caused increase in the PF of Softone in relation to periods of 24 h and seven days (p<0.05), which were not different from each other (p>0.05) (Table 1).

For Trusoft immersed in water, comparison between groups modified by drugs and the control in each evaluation period did not show significant difference for the means of PF (p>0.05), except for the addition of Chx at 14 days, in which a significant increase in mean values was observed (p<0.05) (Table 1). Water immersion did not cause significant change in the PF for Trusoft without modification (control) or modified by Ke or Ny up to 14 days (p>0.05). The addition of Chx caused significant increase in the PF of Trusoft in water for 14 days (p<0.05) compared with periods of 24 h and seven days, which were significantly similar to each other (p>0.05).

In each *S*50 immersion period, the addition of drugs in Softone did not cause significant change in PF in relation to the control (p>0.05). Regardless of the addition of drugs, immersion in S50 for seven days caused reduction in the PF of Softone compared with the 24-h period (p<0.05). There was no difference between the mean values of PF (Table 1) in comparisons between periods of seven and 14 days, and 24 h and 14 days for all study groups of Softone immersed in *S*50 (p>0.05).

Table 1 demonstrates that, when Trusoft was immersed in *S*50, the addition of drugs did not cause significant change in the PF compared with the control for each evaluation period (p>0.05). In control groups and in the groups modified by Ny and Ke, immersion in *S*50 did not cause significant change in the PF for Trusoft during 14 days (p>0.05). There was significant increase in the PF of Trusoft modified by addition of Chx in 14 days of immersion in *S*50 compared with the 7-day period (p<0.05). However, no statistically significant differences were observed between the mean values of PF in periods of 24 h and 14 days (p>0.05). In other words, the porosity of Trusoft at study completion was not altered by Chx in relation to the initial period, which in turn was not different from the control.

Considering the factor “material” in isolation, it was observed that Softone presented significantly higher mean percent PF than Trusoft (p<0.05). For the isolated factor “drug”, the addition of Ny in both materials promoted significantly lower mean percent PF in relation to the other drugs and controls (p<0.05). The control did not present significant difference compared with groups modified by Ke and Chx (p>0.05), which were different from each other (p<0.05), with higher mean PF values for the latter. Considering the isolated factor “solution”, significant reduction in the PF was possible when the materials were immersed in *S*50 compared with water immersion (p<0.05).

## DISCUSSION

Since one objective of temporary resilient liners in the treatment of denture stomatitis is to remove the contact between the contaminated surface in the denture base and infected tissues, it would be ideal to prevent the biofilm formation on them during their lifespan. Because of their greater porosity, temporary materials are easily permeated in depth by nutritive substances present in the oral environment, acting as an incubator for microbial colonization, including *Candida* species^24^. Thus, this study evaluated the effect of addition of antifungals effective for inhibition of *C. albicans* biofilm on the porosity of temporary resilient liners. The null hypothesis was partially accepted, given that the antifungals in most experimental conditions did not adversely affect the porosity of the two short-term resilient liners.

The porosity of acrylic denture base resins has been mainly investigated by naked eye or microscopic analysis^31,32^ and by a method that employs the volume of water absorbed by the material^8,23^. The porosity of temporary acrylic-based soft liners has been scored by microscopic analysis^15,16,21^, a limited method that evaluates the pores only superficially, even when specimens are sectioned^2^. This property is related to the structure of the solid material and is expressed in the presence of empty spaces (pores), i.e., the ratio of free volume inside the polymer. Within this context, a method able to calculate the porosity measuring the specimen mass before and after water immersion is more precise because it evaluates the specimen as a whole^2^. In aqueous medium, the acrylic resin undergoes water sorption by a primary diffusion mechanism; therefore, part of this water occupies spaces present in the material structure and the other part is absorbed, occupying the true pores^2^.

Ideally, a resilient liner should have insoluble components and low water sorption^11,19^. However, during their lifespan, these materials are immersed in saliva, foods, water, and hygiene solutions^7,19,21^. Since this exposure to the aqueous medium concomitantly causes water sorption and loss of plasticizers and other soluble components^14^, the performance and longevity of temporary acrylic-based soft liners depends on the equilibrium of these two mechanisms^4^.

In the present study, compared with the controls, the addition of drugs did not significantly change the percent PF of both temporary soft liners in the different periods of immersion in water or *S*50, except for Chx and Ny in Softone and Chx in Trusoft at 14 days in water. Also, the analysis of the isolated factor “drug” demonstrated that the addition of Ny promoted the lowest mean percent values of PF for both materials in relation to other drugs and controls. The processes of water sorption and lixiviation of components are influenced by the level of diffusion of antifungals through the polymer channels, which in turn depends on the temperature and means of extraction as well as on the molecular weight, particle size, and distribution, solubility and concentration of the drug^5,29^. The level of diffusion of molecules of a drug through a polymer matrix is increased with reduction in its molecular weight^5^. Ke and Chx presented similar molecular weights (531.44 and 625.56 g/mol, respectively), yet lower than Ny (926.11 g/mol). Among the tested antifungals, Chx has the highest water solubility (19 mg/mL), followed by Ny and Ke, which are nearly insoluble in water (4 and 0.017 g/mL, respectively). The low drug solubility in water allows its slow release from the polymer matrix^5^, yielding constant and effective levels for sustained therapies. It has been demonstrated, by a scanning electron microscopy-energy dispersive X-ray spectroscopy (SEM-EDS) analysis, that Ny incorporated to a tissue conditioner displayed small particles (10-50 μm) with random shapes and sizes, yet more uniformly distributed compared with Ke and Chx^29^. The SEM-EDS analysis also showed that Ke specimens presented small spherical particles (10-25 μm) with slight distribution throughout the tissue conditioner matrix. However, Chx specimens exhibited irregular particles up to approximately 50 μm in size randomly dispersed within the matrix. Concerning the concentrations of drugs tested in temporary soft liners in this study, Ny presented the lowest MIC (0.032 g/g), followed by Chx (0.064 g/g) and Ke (0.128 g/g)^6^. Thus, it is reasonable to assume that low MIC, low solubility in water, high molecular weight, and uniform distribution of Ny in the polymer matrix resulted in less formation of empty spaces inside the temporary resilient liners tested, consequently with lower percent PF compared with the other drugs. The favorable results obtained by Ke may be attributed to its small round particles and relative insolubility in water. Finally, the higher solubility of Chx in water and its greater and irregular particles randomly dispersed may have contributed to the increased porosity of Softone and Trusoft at 14 days of water immersion.

Regardless of the addition of drugs, at 14 days of water immersion, there was an increase in the PF of Softone compared with periods of 24 h and seven days, which were similar to each other. Concerning the Trusoft, except when modified by Chx, no significant change in the PF was observed for up to 14 days. Moreover, Softone presented higher mean percent PF compared with Trusoft, regardless of the addition of drugs and storage solution. These results may be assigned to differences in the composition and concentrations of components present in these soft liners. The manufacturer of both short-term resilient materials tested in this study does not specify the composition nor the concentration of plasticizers present in the liquids. According to the literature, Softone and Trusoft are composed of the same polymer (polyethyl methacrylate), which is dissolved in a mixture of phthalate and ethanol plasticizer^16^. Also, both resilient materials present the same powder-liquid ratio^6^. However, it is expected that Softone, as a tissue conditioner, presents greater quantity of plasticizer and alcohol than Trusoft, which is a temporary resilient liner. Clinically, tissue conditioners as Softone should be ideally used for 3-4 days, and their utilization is not recommended for longer than 14 days^16^, which corresponds to the mean lifespan of a temporary resilient liner such as Trusoft^26,27^. According to the manufacturer, because of the low concentration of plasticizers in Trusoft, this material may be clinically used for up to 30 days. Even though both materials present phthalate plasticizers, the liquid of Softone contains dibutyl phthalate and butyl benzoate, while Trusoft contains alkyl phthalate^16^. The molecular weight of dibutyl phthalate present in Softone (194.19 g/mol) is lower than observed for alkyl phthalate of Trusoft (334.44g/mol), and its ethanol concentration (8.3% in volume) is probably higher^16^. It has been reported that the concentration of low molecular weight plasticizer (phthalate) present in Softone is quickly reduced, with greater changes in the first three days, causing loss in its surface integrity^12^. It was demonstrated that ethyl alcohol present in tissue conditioners is completely lixiviated after 24 h of water storage at 37°C. In the first moment, the lixiviation of ethanol is greater than the loss of plasticizer^18^. Even though the tissue conditioners present high initial softness, the fast loss of ethanol and plasticizer results in significant increase in hardness^28^, limiting the lifespan of these materials to a relatively short period. These differences between temporary soft liners analyzed are helpful to explain the stability of porosity of Trusoft in water throughout the immersion period, and the lower percent values of PF in relation to Softone in the different experimental conditions. Also, the increased porosity of Softone after seven days reinforces that its lifespan should ideally be limited to one week.

The present results demonstrated that, when temporary resilient liners were immersed in optimal solution (*S*50), the addition of drugs did not change the PF in relation to the control for each evaluation period. Also, there was significant reduction in the mean percent PF of short-term soft liners immersed in *S*50 in relation to immersion in distilled water. The acrylic resilient materials are known by their slow water absorption over time, due to the polar properties of polymer molecules. These materials do not reach a stage of equilibrium of water absorption even after long periods (up to 6 years)^7^, thus it is difficult to characterize them according to the classic theory of diffusion, as observed for rigid denture base acrylic resins^4^. It is well established in the literature that water absorption causes slight expansion in the polymerized bulk and simultaneously interferes with the entanglement of the polymeric chain, acting as a plasticizer, which alters the physical characteristics of the material^20^. The water absorption of an acrylic resin has been mainly assigned to the hydrophilicity of the polymer, a characteristic that increases according to its number of hydrocarbon groups. Higher level of water absorption has been observed in relation to the lixiviation of components in polyethyl methacrylate materials compared with other polymers such as poly (n-butyl methacrylate)^19^. Some studies also demonstrated that acrylic-based resilient liners present higher values of water sorption than solubility^10,11,19^. In addition to hydrophilicity, other factors related to the polymer, such as density of the matrix and porosity, may interfere with the degree of water absorption. Different from acrylic resins with high degree of polymerization, resilient materials as those tested in this study present low density matrix due to the smaller entanglement of polymeric chains that, despite the increased flexibility, results in greater water diffusion inside it^4^. Simultaneously, the manipulation of acrylic polymers and plasticizers may lead to incorporation of air bubbles, with consequent formation of micropores inside the matrix, facilitating the water sorption^4^. All these findings demonstrate that the primary mechanism involved in sorption is water absorption. Notwithstanding, this property also involves the mechanism of adsorption, whose degree refers to the formation of hydrogen bridges between the water and carboxyl groups, even esterified, being dependent on the chemical composition of the polymer^7^, which is polyethyl methacrylate for both materials analyzed in this study. Considering the great water absorption observed for these polyethyl methacrylate materials, this study employed a method for evaluation of porosity excluding the plasticizer effect of water^23^. Thus, this property was analyzed not only in relation to immersion in distilled water, but also in an optimal storage solution (*S*50), previously determined for each temporary resilient material. The storage of acrylic resin in salt-saturated aqueous solutions, such as *S*50, which allows variation of water activity, allows to estimate the solution in which the plasticizer effect of water would not act (optimal water activity)^25^. It has been demonstrated that the speed of water entry was faster when the materials were stored in distilled water. This highlights the fact that the polymer structure is plasticized by water, yielding spaces between polymeric chains, thus facilitating water entry^3^. This may explain the lower mean percent PF for resilient materials tested when immersed in *S*50 compared with distilled water. Exclusion of the plasticizer effect of water may also be related to the fact that, when the materials were immersed in *S*50, there was no change in PF by the addition of drugs. These results suggest that the porosity values can be unrealistic when acrylic-based materials are stored in distilled water, which reinforces the need of specimen storage in adequate solution.

Within the *in vitro* conditions analyzed in this study, Ny presents the advantage of lowest MIC among all drugs analyzed and also exhibited the smallest percent PF for both temporary resilient liners, thus it may represent a promising protocol for DS treatment. Ny presents the widest spectrum among the antifungals available, being considered as fungicidal. Even though Ke did not alter the porosity of materials tested, the wide antifungal action of Ny associated with a MIC four times lower than Ke are important aspects to be considered when selecting the antifungal for modification of soft liners. However, the prosthetic biofilm is complex and is formed not only by fungi, but also bacteria, which favors the adhesion of fungal cells to the internal surfaces of dentures by co-aggregation^22^. Thus, despite the favorable results observed in this study for Ny and Ke, the addition of Chx to MIC in temporary liners and tissue conditioners should be considered in future studies on alternative therapies for DS treatment for several reasons, including: 1) this drug presents wide spectrum antimicrobial action, reaching bacteria and fungi present in the prosthetic biofilm; 2) Chx presents significant substantivity, resulting in effectiveness in longer evaluation periods^16^; 3) slight changes in the physical and mechanical properties, as observed for Chx in 14 days of water immersion in this study, do not lead to contraindication of the addition of drugs in temporary soft liners, since these are used for short periods^28^; 4) when the materials were immersed in the optimal solution, there was no significant change in the porosity of soft liners modified by Chx up to 14 days.

The results of the present *in vitro* study should be carefully extrapolated to the clinical setting, considering the following restrictions: a) only one trademark of each type of material (tissue conditioner and temporary resilient liner) was evaluated; b) though more objective for the evaluation of porosity, the water sorption method used in this study is limited because it does not offer detailed information on the dimensions and locations of pores within the material^33^; c) clinically, the resilient liners may be subjected to additional stresses such as thermal changes, pH variations and deformation by the occlusal load; d) immersion in distilled water or other solutions do not reproduce the magnitude and rate of changes clinically observed in the properties; e) the loss of compounds that may lixiviate occurs faster because of the oral environment, feeding and hygiene methods adopted by the patient^14^. Therefore, final evaluation of the performance of modified resilient materials can only be determined by clinical studies *in vivo*.

## CONCLUSION

Within the methodological limitations of this *in vitro* study and according to the results obtained, it was concluded that:

The addition of drugs did not significantly change the porosity of both temporary soft liners in the different periods of water immersion, except for Chx and Ny in Softone and Chx in Trusoft at 14 days;

The porosity of short-term resilient liners was not significantly altered by the addition of drugs in up to 14 days of immersion in the optimal storage solution;

In the present experimental conditions, the Softone presented higher porosity compared with Trusoft;

Among the drugs analyzed, the addition of Ny promoted significantly lower porosity for both temporary soft liners.
